# Immediate and long-term changes in infectious diseases in China at the “First-level-response”, “Normalized-control” and “Dynamic-COVID-zero” stages from 2020 to 2022: a multistage interrupted-time-series-analysis

**DOI:** 10.1186/s12889-023-16318-y

**Published:** 2023-07-18

**Authors:** Tianshan Shi, Xiaoshu Zhang, Lei Meng, Donghua Li, Na Jin, Xin Zhao, Hongmiao Zheng, Tingrong Wang, Rui Li, Xiaowei Ren

**Affiliations:** 1 Institute of Epidemiology and Health Statistics, School of Public Health, Lanzhou University, Lanzhou, Gansu China; 2 Gansu Provincial Center for Disease Control and Prevention, Lanzhou, Gansu China

**Keywords:** COVID-19, Infectious disease, Non-pharmaceutical intervention, Dynamic-COVID-zero, Interrupted time series analysis

## Abstract

**Background:**

From January 2020 to December 2022, China implemented “First-level-response”, “Normalized-control” and “Dynamic-COVID-zero” to block the COVID-19 epidemic; however, the immediate and long-term impact of three strategies on other infectious diseases and the difference in their impact is currently unknown. We aim to provide a more comprehensive understanding of the impact of non-pharmacological interventions (NPIs) on infectious diseases in China.

**Methods:**

We collected data on the monthly case count of infectious diseases in China from January 2015 to July 2022. After considering long-term trends using the Cox-Stuart test, we performed the two ratio Z tests to preliminary analyze the impact of three strategies on infectious diseases. Next, we used a multistage interrupted-time-series analysis fitted by the Poisson regression to evaluate and compare the immediate and long-term impact of three strategies on infectious diseases in China.

**Results:**

Compared to before COVID-19, the incidence of almost all infectious diseases decreased immediately at stages 1, 2, and 3; meanwhile, the slope in the incidence of many infectious diseases also decreased at the three stages. However, the slope in the incidence of all sexually transmitted diseases increased at stage 1, the slope in the incidence of all gastrointestinal infectious diseases increased at stage 2, and the slope in the incidence of some diseases such as pertussis, influenza, and brucellosis increased at stage 3. The immediate and long-term limiting effects of “Normalized-control” on respiratory-transmitted diseases were weaker than “First-level-response” and the long-term limiting effects of “Dynamic-COVID-zero” on pertussis, influenza, and hydatid disease were weaker than “Normalized-control”.

**Conclusions:**

Three COVID-19 control strategies in China have immediate and long-term limiting effects on many infectious diseases, but there are differences in their limiting effects. Evidence from this study shows that pertussis, influenza, brucellosis, and hydatid disease began to recover at stage 3, and relaxation of NPIs may lead to the resurgence of respiratory-transmitted diseases and vector-borne diseases.

**Supplementary Information:**

The online version contains supplementary material available at 10.1186/s12889-023-16318-y.

## Background

Coronavirus disease 2019 (COVID-19) is an infectious disease caused by severe acute respiratory syndrome coronavirus 2 (SARS-CoV-2) and poses a serious threat to people’s health [1–3]. After the COVID-19 outbreak, the public health strategies for controlling the COVID-19 epidemic in China included “First-level-response” (January 2020 to April 2020), “Normalized-control” (May 2020 to July 2021), and “Dynamic-COVID-zero” (August 2021 to November 2022) [4]. “First-level-response” is the strongest public health strategy in China, and a series of extremely strict non-pharmacological interventions (NPIs) were adopted at this stage to combat the COVID-19 epidemic [5–7]. Subsequently, the COVID-19 epidemic was gradually controlled in China, and most provinces switched to implementing a less stringent “Normalized-control”, which aims to restore people’s lives to normal [4]. In August 2021, a new strategy called “Dynamic-COVID-zero” was implemented in China to block the spread of the Delta variant and minimize the impact of COVID-19 restrictions on people’s normal lives, and COVID-19 prevention and control was more rapid and precise at this stage [4, 8, 9]. The characteristics, number of COVID-19 cases, and NPIs of the three strategies are shown in Table S1.

NPIs in China during the COVID-19 pandemic have impacted the incidence of many diseases [10–13]. However, previous studies have only studied the impact of NPIs at the previous two stages on infectious diseases, and no studies have studied the impact of “Dynamic-COVID-zero” on infectious diseases. Meanwhile, no research has compared the impact of the three strategies on infectious diseases. Accordingly, it is not clear whether the incidence of infectious diseases will decrease after “Dynamic-COVID-zero” and whether there are differences in the impact of the three strategies on infectious diseases. The World Health Organization (WHO) plans to eliminate or control the epidemic of many infectious diseases such as tuberculosis, viral hepatitis, and tropical diseases by 2030, but the COVID-19 pandemic has interfered with this action [14–16]. It should be noted that “Dynamic-COVID-zero” is the longest-running COVID-19 control strategy in China, and its stringency is closest to that in the post-COVID-19 era. Therefore, supplementing evidence in this field is helpful for fully understanding the impact of COVID-19 restrictions on China’s efforts to eliminate the above infectious diseases, which can guide China to shape public health policy in the future and achieve the WHO goals. On the other hand, some NPIs that can limit the incidence of infectious diseases can be learned for future infectious disease control.

Interrupted time series analysis (ITSA) is a very powerful quasi-experimental design that controls for the effect of the trends before intervention on the time series, thereby allowing us to obtain more robust and reliable results [17]. ITSA can quantitatively evaluate the immediate and long-term changes in outcome variables caused by the interventions to provide more detailed and additional evidence, and its statistical efficacy is even comparable to randomized controlled trials (RCTs) [18]. In this context, this study innovatively used a multi-stage ITSA to evaluate and compare the immediate and long-term effects of three strategies on infectious diseases. We aim to provide a more comprehensive scientific basis for infectious disease control in China in the post-COVID-19 era.

## Materials and methods

### Data collection

Data on the monthly cases of infectious diseases in China from January 2015 to July 2022 were obtained from the infectious disease bulletin of the Chinese National Health Commission (http://www.nhc.gov.cn/jkj/s2907/new_list.shtml). The population data in China were obtained from the World Bank (https://data.worldbank.org.cn/indicator/SP.POP.TOTL?locations=CN). All data used in this study were publicly available aggregate data and did not include human individuals.

### Inclusion of infectious disease

We divided infectious diseases with high incidence from 2015 to 2019 into respiratory-transmitted diseases (measles, tuberculosis, pertussis, scarlet fever, mumps, influenza, rubella, and leprosy), vector-borne diseases (hemorrhagic fever, rabies, Japanese encephalitis [JE], dengue fever, anthrax, brucellosis, malaria, typhus, hydatid disease, Kala-Azar), gastrointestinal infectious diseases (typhoid and paratyphoid [TP], bacterial and amoebic dysentery [BAD], hand foot mouth disease [HFMD], infectious diarrhea), sexually transmitted diseases (acquired immunodeficiency syndrome [AIDS], syphilis, gonorrhea), and infectious diseases with other transmission routes (acute hemorrhagic conjunctivitis [AHC] and viral hepatitis).

### Statistical analysis

We used the Cox-Stuart test to evaluate the long-term trends of infectious diseases from January 2015 to December 2019. Because if the trends of infectious diseases after the interventions (three COVID-19 control strategies) were consistent with their long-term trends before the intervention, then we could not determine whether the intervention had an effect on infectious diseases because the trends after the intervention could be a continuation of their long-term trends before the intervention and not related to the intervention. Conversely, if the trends after the intervention were inconsistent with their long-term trends before the intervention, we then believed that the change in the trends may be caused by the intervention. Furthermore, if the trends after the intervention were consistent with the long-term trends before the intervention, we can next compare the monthly case counts after the intervention with their predicted case counts. If their difference was statistically significant, we also believed that this difference was caused by the intervention. In this study, the predicted case counts were obtained using a seasonal autoregressive integrated moving average (SARIMA) model based on the monthly case counts from January 2015 to December 2019.

Subsequently, we calculated the growth rate of the monthly case counts and used the two ratio Z tests to compare the monthly case counts of infectious diseases at different stages. First, we compared the monthly case counts of infectious diseases at each stage with that in the same period before COVID-19. Moreover, we also compared the monthly case counts of infectious diseases at each stage with that in the same period at the other two stages, respectively. We aimed to evaluate the impact of three strategies on infectious diseases and the differences in their impact. The calculation formula for the growth rate was:

$$\begin{array}{l}growth\,rate\, = \,\frac{{number\,of\,cases(later) - number\,of\,cases(previous)}}{{number\,of\,cases(previous)}}\\\times \,100\% \end{array}$$

After the above comparison, we used the monthly case counts from January 2017 to July 2022 to conduct the ITSA for infectious diseases affected by COVID-19 control strategies. The data points used in the ITSA were sufficient and we also adjusted the seasonality using Fourier terms to improve the linear trend of the time series, so this study met the applicable criteria of ITSA [19]. In our study, ITSA took three strategies as interventions. The first intervention was “First-level-response” which was implemented for only a few days in January 2020, so we took February 2020 as the first intervention time point in order not to underestimate the impact of “First-level-response” on infectious diseases. The second and third interventions were “Normalized-control” and “Dynamic-COVID-zero”. The implementation periods of the three strategies were named stages 1, 2, and 3. Next, we modeled directly based on the monthly case counts using the Poisson regression. Then, we used the population (log-transformed) as an offset variable to transform back to rates [20]. After using the Poisson regression to fit the ITSA, we calculated the incidence rate ratio (IRR) according to the regression coefficient (*β*) obtained from the Poisson regression and took it as effect estimate. Detailed information, parameter explanations, and IRR calculation formula of the ITSA can be found in Supplementary Material.

Noteworthy, the number of data points may affect the robustness of the time series analysis results, so we again used the same method to re-construct the ITSA for sensitivity analysis based on the monthly case counts of infectious diseases from January 2015 to July 2022. After sensitivity analysis, the robustness of results was considered excellent if the IRRs for the majority of parameters did not show significant differences and the main findings did not change.

The Cox-Stuart test, two ratio Z tests, and SARIMA model were performed using R software (version 4.0.3, R Development Core Team 2020), ITSA and figure production was performed using Stata software (version 15.0, StataCorp, College Station, TX, USA). All testing was two-sided with significance determined at *P* < 0.05.

## Results

### Preliminary analysis

Compared to January to April 2019, the monthly case counts of almost all infectious diseases at stage 1 were lower but the trends of measles and malaria were consistent with the long-term trends (Table 1). However, the monthly case counts of measles (N_predict_=271, growth rate = − 63.10%, *P* < 0.05) and malaria (N_predict_=231, growth rate = − 32.90%, *P* < 0.05) were lower than the predicted case counts. Compared to May 2018 to July 2019, the monthly case counts of most infectious diseases remained lower but the monthly case counts of brucellosis were higher at stage 2. Compared to August 2018 to July 2019, the monthly case counts of almost all infectious diseases were lower but the monthly case counts of pertussis, influenza, and brucellosis were higher at stage 3.

**Table 1:** Impact of “First-level-response”, “Normalized-control” and “Dynamic-zero-COVID” on infectious diseases in China

Diseases	Long-term trends	2019*	2020*	Growth rate (%)	*P*-value	2018–2019#	2020–2021#	Growth rate (%)	*P*-value	2018–2019##	2021–2022##	Growth rate (%)	*P*-value
**Respiratory-transmitted diseases**													
Measles	Decreasing	269	100	-62.83	< 0.001	326	87	-73.31	< 0.001	281	80	-71.53	< 0.001
Tuberculosis	No	90188	67932	-24.68	< 0.001	91791	74148	-19.22	< 0.001	90655	61351	-32.32	< 0.001
Pertussis	Increasing	2032	808	-60.24	< 0.001	2382	292	-87.74	< 0.001	2429	2590	6.63	0.032
Scarlet fever	Increasing	5878	1955	-66.74	< 0.001	7205	1880	-73.91	< 0.001	6769	2137	-68.43	< 0.001
Influenza	Increasing	393775	270697	-31.26	< 0.001	150324	21912	-85.42	< 0.001	183392	203695	11.07	< 0.001
Mumps	Increasing	20952	9344	-55.40	< 0.001	25244	10631	-57.89	< 0.001	23992	9864	-58.89	< 0.001
Rubella	No	3027	455	-84.97	< 0.001	2200	119	-94.59	< 0.001	2627	107	-95.93	< 0.001
Leprosy	Decreasing	47	35	-25.53	0.183	47	39	-17.02	0.380	45	31	-31.11	0.105
**Vector-borne diseases**													
Hemorrhagic fever	No	813	508	-37.52	< 0.001	986	683	-30.73	< 0.001	966	755	-21.84	< 0.001
Rabies	Decreasing	24	15	-37.50	0.148	32	17	-46.88	0.031	32	12	-62.50	0.002
JE	No	4	3	-25.00	0.704	138	22	-84.06	< 0.001	145	19	-86.90	< 0.001
Dengue fever	Increasing	109	28	-74.31	< 0.001	525	48	-90.86	< 0.001	625	2	-99.68	< 0.001
Anthrax	No	13	10	-23.08	0.529	30	23	-23.33	0.330	31	35	12.90	0.632
Brucellosis	No	3299	3062	-7.18	0.002	3886	5768	48.43	< 0.001	3639	6359	74.75	< 0.001
Malaria	Decreasing	210	155	-26.19	0.004	220	72	-67.27	< 0.001	216	49	-77.31	< 0.001
Typhus	No	53	48	-9.43	0.614	92	104	13.04	0.404	92	122	32.61	0.043
Hydatid disease	No	421	247	-41.33	< 0.001	404	326	-19.31	0.003	411	272	-33.82	< 0.001
Kala-azar	Decreasing	16	20	25.00	0.508	16	20	25.00	0.511	15	24	60.00	0.153
**Gastrointestinal infectious diseases**													
TP	No	699	413	-40.92	< 0.001	973	652	-32.99	< 0.001	938	574	-38.81	< 0.001
BAD	No	4345	2952	-32.06	< 0.001	7990	5172	-35.27	< 0.001	7158	3561	-50.25	< 0.001
HFMD	No	80377	9372	-88.34	< 0.001	229939	108155	-52.96	< 0.001	188442	78163	-58.52	< 0.001
Infectious diarrhea	Increasing	113283	68505	-39.53	< 0.001	108267	114739	5.98	< 0.001	109036	88905	-18.46	< 0.001
**Sexually transmitted diseases**													
AIDS	Increasing	4910	3915	-20.26	< 0.001	5961	5296	-11.16	< 0.001	6061	4933	-18.61	< 0.001
Syphilis	Increasing	44156	37250	-15.64	< 0.001	46524	46291	-0.50	0.207	46351	43474	-6.21	< 0.001
Gonorrhea	No	9108	5677	-37.67	< 0.001	10763	10469	-2.73	0.024	10432	9706	-6.96	< 0.001
**Infectious diseases with other transmission routes**													
AHC	No	2652	2013	-24.10	< 0.001	3567	2469	-30.78	< 0.001	3461	2291	-33.81	< 0.001
Viral hepatitis	No	133043	102314	-23.10	< 0.001	129733	127213	-1.94	< 0.001	129482	127861	-1.25	< 0.001

JE: Japanese encephalitis; TP: Typhoid and paratyphoid; BAD: Bacterial and amoebic dysentery; HFMD: Hand-foot-and-mouth disease; AIDS: Acquired immunodeficiency syndrome; AHC: Acute hemorrhagic conjunctivitis.

* indicates the number of monthly cases of infectious diseases from January 2019 to April 2019 and from January 2020 to April 2020, respectively.

# indicates the number of monthly cases of infectious diseases from May 2018 to July 2019 and from May 2020 to July 2021, respectively.

## indicates the number of monthly cases of infectious diseases from August 2018 to July 2019 and from August 2021 to July 2022, respectively.

Compared to stage 1, the monthly case counts of some vector-borne diseases (brucellosis and hydatid disease), gastrointestinal infectious diseases (HFMD and infectious diarrhea), and sexually transmitted diseases (syphilis and gonorrhea) were higher at stages 2 and 3, see Table 2. Compared to stage 2, the monthly case counts of almost all infectious diseases were lower but the monthly cases of pertussis, influenza, and brucellosis were higher at stage 3.

**Table 2:** Differences in the impact of “First-level-response”, “Normalized-control” and “Dynamic-zero-COVID” on infectious diseases in China

Diseases	Long-term trends	2020*	2021*	Growth rate (%)	*P*-value	2020*	2022*	Growth rate (%)	*P*-value	2020–2021#	2021–2022#	Growth rate (%)	*P*-value
**Respiratory-transmitted diseases**													
Measles	Decreasing	100	61	-39.00	0.002	100	52	-48.00	< 0.001	86	80	-6.98	0.641
Tuberculosis	No	67932	70397	3.63	< 0.001	67932	62147	-8.52	< 0.001	71731	61351	-14.47	< 0.001
Pertussis	Increasing	808	212	-73.76	< 0.001	808	3023	274.13	< 0.001	317	2590	717.03	< 0.001
Scarlet fever	Increasing	1955	2025	3.58	0.272	1955	1927	-1.43	0.646	2182	2137	-2.06	0.490
Influenza	Increasing	270697	19943	-92.63	< 0.001	270697	125040	-53.81	< 0.001	23555	203695	764.76	< 0.001
Mumps	Increasing	9344	8144	-12.84	< 0.001	9344	7759	-16.96	< 0.001	10635	9864	-7.25	< 0.001
Rubella	No	455	91	-80.00	< 0.001	455	80	-82.42	< 0.001	125	107	-14.40	0.237
Leprosy	Decreasing	35	40	14.29	0.565	35	32	-8.57	0.713	37	31	-16.22	0.466
**Vector-borne diseases**													
Hemorrhagic fever	No	508	465	-8.46	0.166	508	447	-12.01	0.048	686	755	10.06	0.070
Rabies	Decreasing	15	15	0.00	1.000	15	9	-40.00	0.220	17	12	-29.41	0.353
JE	No	3	1	-66.67	0.317	3	3	0.00	1.000	25	19	-24.00	0.365
Dengue fever	Increasing	28	3	-89.29	< 0.001	28	1	-96.43	< 0.001	57	2	-96.49	< 0.001
Anthrax	No	10	14	40.00	0.415	10	14	40.00	0.415	25	35	40.00	0.197
Brucellosis	No	3062	5417	76.91	< 0.001	3062	5716	86.68	< 0.001	5719	6359	11.19	< 0.001
Malaria	Decreasing	155	66	-57.42	< 0.001	155	30	-80.65	< 0.001	76	49	-35.53	0.016
Typhus	No	48	56	16.67	0.434	48	57	18.75	0.381	104	122	17.31	0.232
Hydatid disease	No	247	334	35.22	< 0.001	247	295	19.43	0.040	331	272	-17.82	0.016
Kala-azar	Decreasing	20	19	-5.00	0.872	20	25	25.00	0.457	19	24	26.32	0.446
**Gastrointestinal infectious diseases**													
TP	No	413	425	2.91	0.682	413	379	-8.23	0.225	624	574	-8.01	0.148
BAD	No	2952	3172	7.45	0.005	2952	2409	-18.39	< 0.001	4683	3561	-23.96	< 0.001
HFMD	No	9372	65528	599.19	< 0.001	9372	35100	274.52	< 0.001	132114	78163	-40.84	< 0.001
Infectious diarrhea	Increasing	68505	143476	109.44	< 0.001	68505	92610	35.19	< 0.001	116952	88905	-23.98	< 0.001
**Sexually transmitted diseases**													
AIDS	Increasing	3915	4386	12.03	< 0.001	3915	3832	-2.12	0.338	5076	4933	-2.82	0.151
Syphilis	Increasing	37250	43372	16.43	< 0.001	37250	40190	7.89	< 0.001	45890	43474	-5.26	< 0.001
Gonorrhea	No	5677	9922	74.78	< 0.001	5677	8240	45.15	< 0.001	10752	9706	-9.73	< 0.001
**Infectious diseases with other transmission routes**													
AHC	No	2013	2218	10.18	0.002	2013	2097	4.17	0.194	2415	2291	-5.13	0.070
Viral hepatitis	No	102314	126792	23.92	< 0.001	102314	127001	24.13	< 0.001	127585	127861	0.22	0.615

JE: Japanese encephalitis; TP: Typhoid and paratyphoid; BAD: Bacterial and amoebic dysentery; HFMD: Hand-foot-and-mouth disease; AIDS: Acquired immunodeficiency syndrome; AHC: Acute hemorrhagic conjunctivitis.

* indicates the number of monthly cases of infectious diseases from January 2020 to April 2020, from January 2021 to April 2021, and from January 2022 to April 2022, respectively.

# indicates the number of monthly cases of infectious diseases from August 2020 to July 2021 and from August 2021 to July 2022, respectively.

### ITSA of respiratory-transmitted diseases

**Fig. 1:**
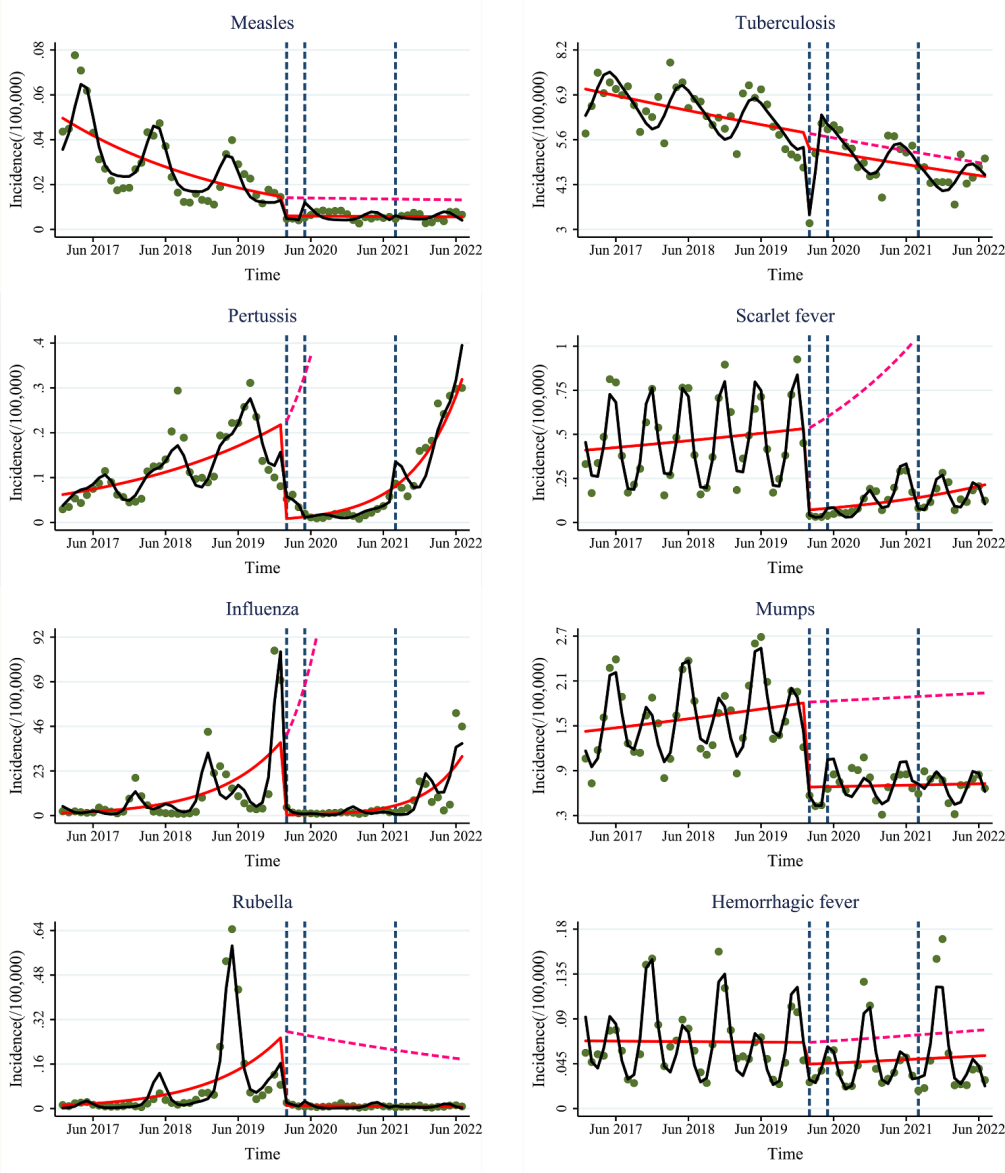
Trends in the incidence of respiratory-transmitted and vector-borne diseases at the “First-level-response”, “Normalized-control”, and “Dynamic-zero-COVID” stages. Green dots: the monthly incidence of infectious diseases; blue dashed lines: the three strategies; red solid line: predicted trend based on the unadjusted regression model; red dashed line: predicted by counterfactual based on the unadjusted regression model; black solid line: predicted trend based on the seasonally adjusted regression model

Compared with the pre-COVID-19 period, the incidence of all respiratory-transmitted diseases decreased immediately and only the slope in the tuberculosis incidence increased at stage 1, see Fig. 1 and S1. “First-level-response” had strong immediate and long-term limiting effects on respiratory-transmitted diseases. After entering stage 2, the incidence of almost all respiratory-transmitted diseases decreased immediately and their slopes also decreased compared with the pre-COVID-19 period, however, the incidence of many respiratory-transmitted diseases increased immediately and their slopes also increased compared with stage 1. “Normalized-control” still had immediate and long-term effects on respiratory-transmitted diseases, but were weaker than “First-level-response”. After entering stage 3, the incidence of a few respiratory-transmitted diseases still decreased immediately and their slopes also decreased compared with the pre-COVID-19 period; however, the incidence of many respiratory-transmitted diseases increased immediately and their slope also increased compared with stage 2. “Dynamic-COVID-zero” had weaker immediate and long-term effects on respiratory-transmitted diseases than “Normalized-control”.

### ITSA of vector-borne diseases

**Fig. 2:**
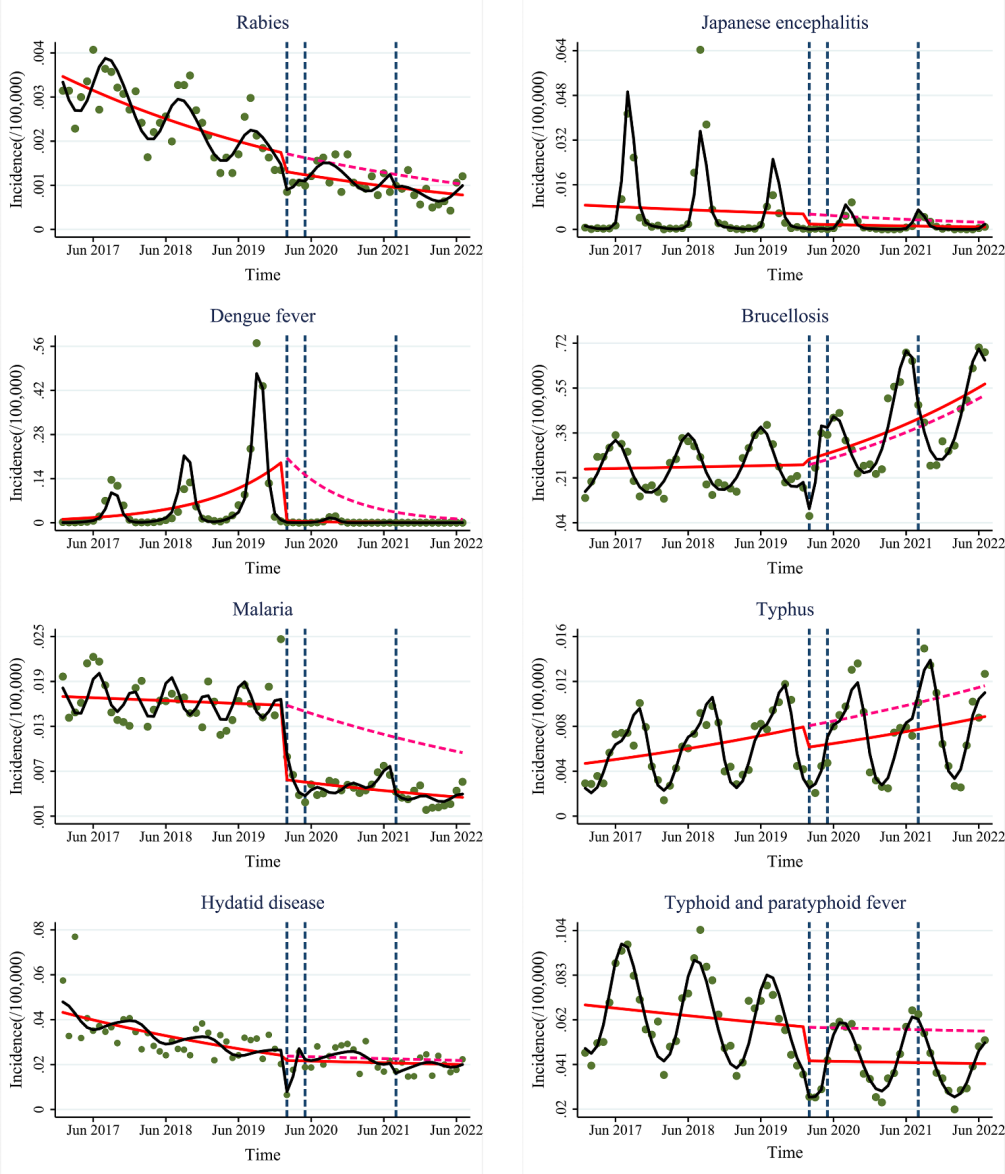
Trends in the incidence of vector-borne and gastrointestinal diseases at the “First-level-response”, “Normalized-control”, and “Dynamic-zero-COVID” stages. Green dots: the monthly incidence of infectious diseases; blue dashed lines: the three strategies; red solid line: predicted trend based on the unadjusted regression model; red dashed line: predicted by counterfactual based on the unadjusted regression model; black solid line: predicted trend based on the seasonally adjusted regression model

Compared with the pre-COVID-19 period, the incidence of most vector-borne diseases decreased immediately but the slope in the incidence of some vector-borne diseases increased at stage 1, see Figs. 1-2 and Figs S1-S2. “First-level-response” had strong immediate but weaker long-term limiting effects on vector-borne diseases. After entering stage 2, the incidence of many vector-borne diseases decreased immediately compared with the pre-COVID-19 period and stage 1. “Normalized-control” still had immediate limiting effects on some vector-borne diseases. Compared with the pre-COVID-19 period, the incidence of almost all vector-borne diseases still decreased immediately but the slope in the incidence of brucellosis and hydatid disease increased at stage 3. Similarly, the incidence of most vector-borne diseases decreased immediately at stage 3 compared with stage 2. “Dynamic-COVID-zero” still had immediate limiting effects on most vector-borne diseases and was even stronger than “Normalized-control”. However, the long-term limiting effects of “Normalized-control” and “Dynamic-COVID-zero” on brucellosis and hydatid disease were weaker.

### ITSA of other infectious diseases

**Fig. 3:**
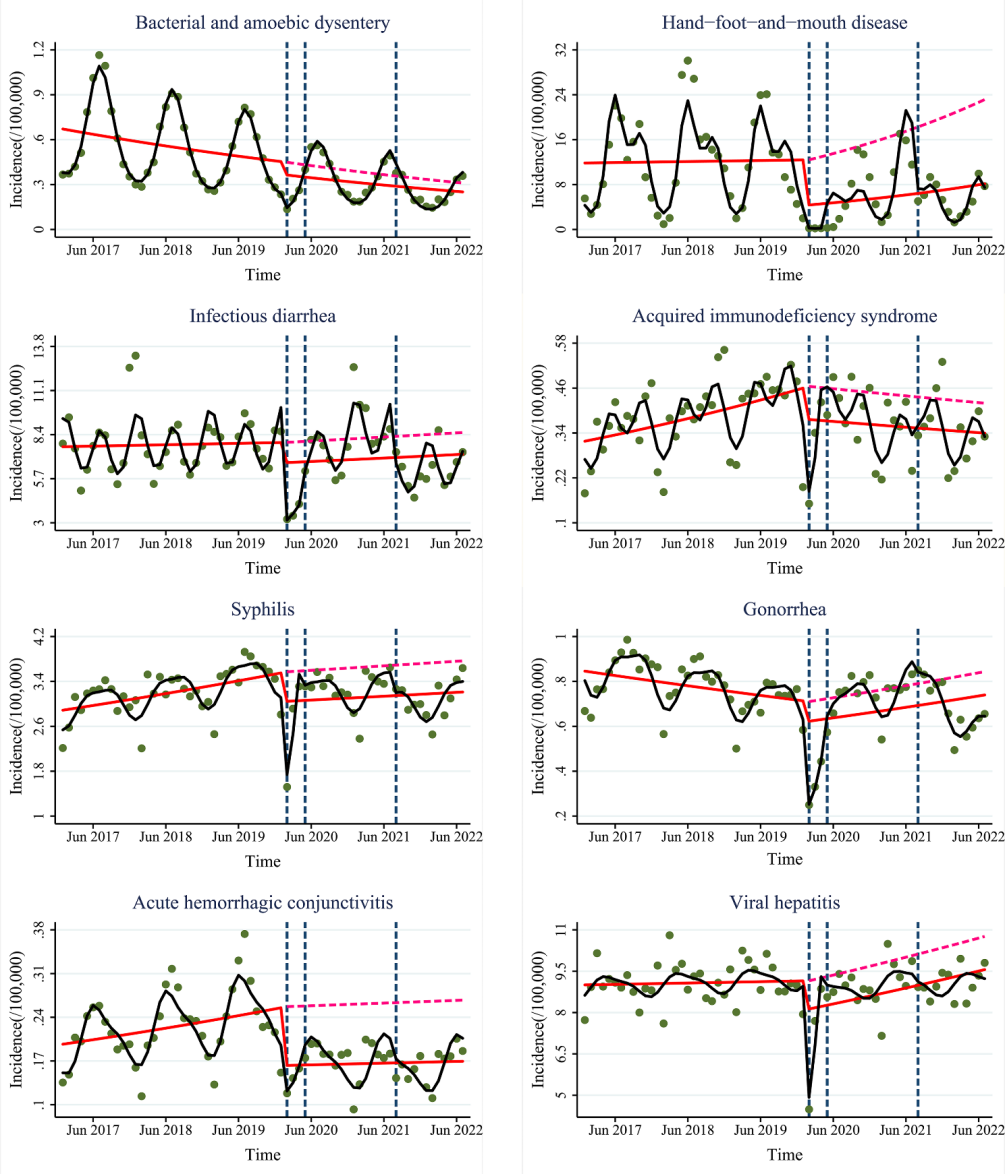
Trends in the incidence of other infectious diseases at the “First-level-response”, “Normalized-control”, and “Dynamic-zero-COVID” stages. Green dots: the monthly incidence of infectious diseases; blue dashed lines: the three strategies; red solid line: predicted trend based on the unadjusted regression model; red dashed line: predicted by counterfactual based on the unadjusted regression model; black solid line: predicted trend based on the seasonally adjusted regression model

Compared with the pre-COVID-19 period, the incidence of all gastrointestinal infectious diseases, sexually transmitted diseases, and infectious diseases with other transmission routes decreased immediately but the slope of all sexually transmitted diseases increased at stage 1, see Figs. 2-3 and Figs S2-S3. “First-level-response” had strong immediate limiting effects on gastrointestinal infectious diseases, sexually transmitted diseases, and infectious diseases with other transmission routes but had weaker long-term limiting effects on sexually transmitted diseases.

After entering stage 2, the incidence of many gastrointestinal infectious diseases, all sexually transmitted diseases, and all infectious diseases with other transmission routes decreased immediately and the slope of many sexually transmitted diseases also decreased compared with the pre-COVID-19 period; the incidence of many gastrointestinal infectious diseases increased immediately and the slope of all sexually transmitted diseases decreased compared with stage 1. “Normalized-control” still had immediate limiting effects on gastrointestinal infectious diseases, sexually transmitted diseases, and infectious diseases with other transmission routes, but were weaker than “First-level-response”. Meanwhile, “Normalized-control” had stronger long-term limiting effects on sexually transmitted diseases.

After entering stage 3, the incidence of all gastrointestinal infectious diseases, sexually transmitted diseases, and infectious diseases with other transmission routes decreased immediately and the slope of many gastrointestinal infectious diseases also decreased compared with the pre-COVID-19 period. Similarly, the incidence of many gastrointestinal infectious diseases, sexually transmitted diseases, and infectious diseases with other transmission routes decreased immediately and the slope of many gastrointestinal infectious diseases also decreased compared with stage 2. “Dynamic-COVID-zero” still had immediate limiting effects on gastrointestinal infectious diseases, sexually transmitted diseases, and infectious diseases with other transmission routes, and were stronger than “Normalized-control”. Meanwhile, “Dynamic-COVID-zero” had stronger long-term limiting effects on gastrointestinal infectious diseases than “Normalized-control”.

### Sensitivity analysis

We used January 2015 to January 2020 as the pre-COVID-19 period and then fitted the ITSA for sensitivity analysis. Tables S2-S7 demonstrate that the results of the sensitivity analysis were similar to the findings of the primary ITSA, and increasing the time series before COVID-19 in the ITSA did not significantly affect the results of this study. In detail, in the sensitivity analysis, we found that only a few of the IRRs for some parameters of infectious diseases with low incidence such as measles were slightly changed, while the IRRs for parameters of the vast majority of infectious diseases were virtually unchanged, and the conclusions of the two ITSAs were not substantially different. In other words, the immediate and long-term impact of the three strategies on infectious diseases derived from the primary ITSA and sensitivity analysis were consistent. Thus, the robustness of our results was excellent.

## Discussion

COVID-19 poses a significant threat to the life and health of the population worldwide, and effective and rigorous strategies for COVID-19 control are critical to block the COVID-19 epidemic [21, 22]. Studies have confirmed that NPIs in many countries have impacted the COVID-19 pandemic, but have also had some impact on other infectious diseases [10, 23–25]. In this study, we found that the implementation of “First-level-response” had significant limiting effects on almost all infectious diseases. When the COVID-19 control strategy was adjusted from “First-level-response” to strategies with lower strictness, NPIs still had a limiting effect on many infectious diseases. In particular, NPIs had more obvious limiting effects on infectious diseases with a high incidence. However, pertussis, influenza, and brucellosis started to rapidly recover at stage 3, and NPIs with low stringency had no limiting effects on their incidence. In general, “First-level-response” had the strongest limiting effects on infectious diseases. “Dynamic-COVID-zero” had stronger limiting effects on gastrointestinal infectious diseases and sexually transmitted diseases but had weaker limiting effects on respiratory-transmitted diseases compared to “Normalized-control”. The incidence of pertussis and influenza increased sharply at stage 3. Our evidence suggests that the risk of respiratory-transmitted disease outbreaks will increase when the stringency of NPIs decreases.

### Immediate and long-term effects of three strategies on respiratory-transmitted diseases

“First-level-response” reduced immediately the incidence of almost all respiratory-transmitted diseases, and the trends in their incidence at stage 1 were also decreasing, which may be related to the following reasons. Firstly, mainland China required residents to live and work at home and closed schools and entertainment venues as well as banned large gatherings at stage 1, so the movement and concentration of the population were immediately and severely restricted at this stage [26, 27]. These initiatives would create enough social distance between people to cut off the spread of respiratory-transmitted diseases, resulting in a decrease in the incidence of respiratory-transmitted diseases, which could be confirmed by a study in the United States [28]. Furthermore, increased awareness of personal protection has caused a decrease in the incidence of respiratory-transmitted diseases, such as environmental disinfection, wearing masks, and wearing protective gloves can kill pathogens, block transmission routes, and protect susceptible individuals, thereby reducing the incidence of respiratory-transmitted diseases [29]. However, the slope in the incidence of tuberculosis was increasing at stage 1, and the long-term limiting effect of “First-level-response” on tuberculosis is weaker. This may be related to the fact that with the effective control of COVID-19, the stringency of NPIs was decreased over time at stage 1. Moreover, the trend in the incidence of tuberculosis increased at stage 1 may also be associated with its chronic nature. That is, the progress of tuberculosis patients is slow, and patients may be restricted from seeking medical care due to community closures and traffic restrictions when they develop mild symptoms of tuberculosis at the start of stage 1, thus delaying the diagnosis of tuberculosis [30, 31]. Noteworthy, patients may refuse to actively seek medical care for fear of developing COVID-19, thus accumulating cases until they are diagnosed at the end of stage 1, which causes the reported cases to increase [32, 33].

Using the pre-COVID-19 period as a reference, the incidence of almost all respiratory-transmitted diseases also decreased immediately at stage 2. This is because although the stringency of NPIs has declined and social activities are gradually returning to normal, people’s mask wear remained high and the number of travel activities and the size of gatherings were limited at this stage, so these measures remained effective in reducing the incidence of respiratory-transmitted infections. However, compared with stage 1, the incidence of many respiratory-transmitted diseases increased immediately at stage 2, and their trends also increased at this stage. Which may be related to the decline in the strictness of COVID-19 control measures.

At stage 3, the incidence of most respiratory-transmitted diseases was lower but the trends and incidence of pertussis and influenza were increasing. Although the stringency of NPIs declined at stage 3, it was still higher than that during the pre-COVID-19 period, so “Dynamic-COVID-zero” still has a little limiting effect on respiratory-transmitted diseases. Moreover, the motivation of people to actively seek medical care gradually increased with the decreasing stringency of NPIs at stage 3, so the number of patients that developed tuberculosis and other infectious diseases at stage 2 was delayed and accumulated to be diagnosed at stage 3 was very little, which also caused the incidence of some infectious diseases at stage 3 was lower. However, it should be noted that the limiting effects of “Dynamic-COVID-zero” on respiratory-transmitted diseases (especially pertussis and influenza) may be weaker than the first two strategies. We believed that the possible causes of this phenomenon are the stringency of NPIs, the geographic regions affected by NPIs, and the number of people affected by NPIs at this stage were lower than at the previous two stages [4, 8]. We inferred that NPIs with low stringency may not limit the increase in the incidence of pertussis and influenza. In the post-COVID-19 era, we need to be alert and prepared for the resurgence of pertussis and influenza at any time.

### Immediate and long-term effects of three strategies on vector-borne diseases

Compared with the pre-COVID-19 period, three COVID-19 control strategies can effectively reduce the incidence of many vector-borne diseases (such as dengue fever, brucellosis, malaria, and hydatid disease) in the short term. This is because bats have been considered as the host of COVID-19 at the early stage, which has led to increased vigilance of people against wildlife and reduced the risk of contracting vector-borne diseases from contact with wildlife [34, 35]. Moreover, transportation restrictions, community lockdowns, and fear of COVID-19 can reduce the incentive for people to seek active medical care, thereby leaving many diseases undiagnosed [36], which can also reduce the reported incidence of vector-borne diseases. Furthermore, dengue fever is a common imported infectious disease, and even small numbers of imported dengue cases can trigger locally acquired infections and even local outbreaks [37]. A study in Guangdong, a region with a high prevalence of dengue fever in China, found that international flight traffic to Guangdong decreased by 83.1% in 2020, while the number of dengue cases decreased significantly at the same time [38]. Thus, border restrictions and declines in human mobility during the COVID-19 pandemic in China could significantly reduce the incidence of imported infectious diseases such as dengue fever [37, 39]. However, the trends of brucellosis at stages 2 and 3 and hydatid disease at stage 3 were increasing. This may be related to the fact that NPIs in rural areas are not as strict as that in cities, and NPIs cannot significantly reduce the contact between farmers and livestock. On the other side, although the animal husbandry trade in mainland China is limited after the COVID-19 outbreak. However, to protect people’s daily diet, the impact of NPIs on the animal husbandry trade and meat trade may be far less than that of other industries, which can lead to an increase in the incidence of brucellosis and hydatid disease.

### Immediate and long-term effects of three strategies on other infectious diseases

Three COVID-19 control strategies can reduce immediately the incidence of most gastrointestinal infectious diseases, sexually transmitted diseases, and infectious diseases with other transmission routes. This is because the closure of restaurants, increased environmental disinfection, restricted close contact, and personal cleaning can affect the spread of gastrointestinal infectious diseases [40, 41]. In addition, closing schools and early childhood care facilities can reduce significantly the incidence of HFMD [42]. However, the immediate limiting effects of “Normalized-control” on some gastrointestinal infectious diseases (such as TP, HFMD, and infectious diarrhea) and gonorrhea were weaker than “First-level-response”. This phenomenon may be related to the reopening of educational institutions and restaurants and the recovery of social distance at this stage. The incidence of sexually transmitted diseases and viral hepatitis decreased immediately at stage 1, which was associated with a change in the sexual lifestyle during the COVID-19 pandemic. The home isolation order restricted extramarital sex and commercial sex transactions, reducing the spread chances of sexually transmitted diseases [25, 43]. However, after entering stage 2, the incidence of gonorrhea increased gradually over time, which is closely related to the lifting of the blockade and the resumption of social activities. Moreover, gonorrhea is an acute disease that is less likely to have a delay in diagnosis than chronic infectious diseases, which is one reason why the incidence of chronic infectious diseases such as AIDS and syphilis did not increase rapidly at this stage. Although three strategies can limit infectious diarrhea, syphilis, and viral hepatitis in the short term, their incidence showed increasing trends at stages 1, 2, and 3.

### Limitations

Our study classifies infectious diseases by transmission routes and also takes into account the long-term trends and seasonality of infectious diseases, providing a comprehensive evaluation of the impact of three strategies on infectious diseases in China. However, there are some limitations. First, our study used summary data on infectious diseases, so we could not understand the impact of three COVID-19 control strategies on infectious diseases in different regions and different age groups. Moreover, the decrease in the number of reported cases of some infectious diseases may be caused by a decrease in surveillance sensitivity during the COVID-19 epidemic, which does not imply a decrease in the true number of infectious disease cases. Next, this study is a non-experimental study that describes more of a correlation than a causal relationship between the prevention and control measures of COVID-19 and the incidence of infectious diseases. Finally, this study does not include potential influences such as meteorological factors in the ITSA.

## Electronic supplementary material

Below is the link to the electronic supplementary material.

Supplementary Material 1

Supplementary Material 2

Supplementary Material 3

Supplementary Material 4

## Data Availability

Data on the monthly cases of legally reported infectious diseases in China are obtained from the infectious disease bulletin of the Chinese National Health Commission (http://www.nhc.gov.cn/jkj/s2907/new_list.shtml). The population data in China were obtained from the World Bank (https://data.worldbank.org.cn/indicator/SP.POP.TOTL?locations=CN). In addition, we provide the data used in this study in Supplementary Data.
