# Supplementary material for: Immediate and long-term changes in infectious diseases in China at the “First-level-response”, “Normalized-control” and “Dynamic-COVID-zero” stages from 2020 to 2022: a multistage interrupted-time-series-analysis
Source: BMC Public Health. 2023 Jul 18;23:1381. doi: 10.1186/s12889-023-16318-y (PMC10354895; doi:10.1186/s12889-023-16318-y)
Supplement: Supplementary file 1 — Supplementary Material 1 [file 12889_2023_16318_MOESM1_ESM.docx]

**Immediate and long-term changes in infectious diseases in China at the “First-level-response”, “Normalized-control” and “Dynamic-COVID-zero” stages from 2020 to 2022: A multistage interrupted-time-series-analysis**

Tianshan Shi^1^, Xiaoshu Zhang^2^, Lei Meng^2^, Donghua Li^1^, Na Jin^2^, Xin Zhao^1^, Hongmiao Zheng^1^, Tingrong Wang^1^, Rui Li^1^, Xiaowei Ren^1*^

^1^ Institute of Epidemiology and Health Statistics, School of Public Health, Lanzhou University, Lanzhou, Gansu, China.

^2^ Gansu Provincial Center for Disease Control and Prevention, Lanzhou, Gansu, China.

***Correspondence:** [renxw@lzu.edu.cn](mailto:renxw@lzu.edu.cn)

Tianshan Shi and Xiaoshu Zhang contributed equally to this study.

**Detailed information and parameter explanations of ITSA modeling**

In the ITSA of this study, the number of monthly cases of infectious diseases was taken as the outcome variable *Y*; *X_1_* was the time variable (with values of 1, 2, 3, …, n in order by time); *X_2_* was an intervention variable which represented the introduction of “First-level-response” (0 prior to February 2020, 1 from February 2020); *X_3_* was the time variable after the introduction of “First-level-response” (0 prior to February 2020, with values of 0, 1, 2, …, n in order by time from February 2020); *X_4_* was an intervention variable which represented the introduction of “Normalized-control” (0 prior to May 2020, 1 from May 2020); *X_5_* was the time variable after the introduction of “Normalized-control” (0 prior to May 2020, with values of 0, 1, 2, …, n in order by time from May 2020); *X_6_* was an intervention variable which represented the introduction of “Dynamic-COVID-zero” (0 prior to August 2021, 1 from August 2021); *X_7_* was the time variable after the introduction of “Dynamic-COVID-zero” (0 prior to August 2021, with values of 0, 1, 2, …, n in order by time from August 2021) [19-22]. The ITSA model was as follows:

$$Y_{t}=\beta_{0}+\beta_{1}X_{1}+\beta_{2}X_{2}+\beta_{3}X_{3}+\beta_{4}X_{4}+\beta_{5}X_{5}+\beta_{6}X_{6}+\beta_{7}X_{7}$$

*β_0_* represented the baseline level of the incidence of infectious diseases; *β_1_* represented the trend in the incidence of infectious diseases in the pre-COVID-19 period. We took the last intervention period as a reference, *β_2_* was the level change in the incidence of infectious diseases at stage 1 and represented the immediate impact of “First-level-response” on infectious diseases; *β_3_* was the slope change in the incidence of infectious diseases at stage 1 and represented the long-term impact of “First-level-response” on infectious diseases; *β_4_* was the level change in the incidence of infectious diseases at stage 2 and represented the immediate impact of “Normalized-control” on infectious diseases; *β_5_* was the slope change in the incidence of infectious diseases at stage 2 and represented the long-term impact of “Normalized-control” on infectious diseases; *β_6_* was the level change in the incidence of infectious diseases at stage 3 and represented the immediate impact of “Dynamic-COVID-zero” on infectious diseases; *β_7_* was the slope change in the incidence of infectious diseases at stage 3 and represented the long-term impact of “Dynamic-COVID-zero” on infectious diseases [20-22].

We took the pre-COVID-19 period (January 2017 to January 2020) as a reference, *β_2_*+*β_4_* was the level change in the incidence of infectious diseases at stage 2 and represented the immediate impact of “Normalized-control” on infectious diseases; *β_2_*+*β_4_*+*β_6_* was the level change in the incidence of infectious diseases at stage 3 and represented the immediate impact of “Dynamic-COVID-zero” on infectious diseases; *β_3_*+*β_5_* was the slope change in the incidence of infectious diseases at stage 2 and represented the long-term impact of “Normalized-control” on infectious diseases; *β_3_+β_5_*+*β_7_* was the slope change in the incidence of infectious diseases at stage 3 and represented the long-term impact of “Dynamic-COVID-zero” on infectious diseases. *β_1_*+*β_3_* was the trend in the incidence of infectious diseases at stage 1; *β_1_*+*β_3_*+*β_5_* was the trend in the incidence of infectious diseases at stage 2; *β_1_*+*β_3_*+*β_5_*+*β_7_* was the trend in the incidence of infectious diseases at stage 3 [20, 21]. In this study, we used the incidence rate ratio (IRR) and its 95% confidence interval (95% CI) as effect estimates, and the calculation formula was: IRR = exp (*β_j_*).

In the ITSA, the level change was the difference between the predicted value of the post-intervention regression equation at the first intervention point and the predicted value of the pre-intervention regression equation extended to the intervention point (the difference compared to the counterfactual); the amount of slope change was the difference between the slopes of the two regression equations before and after the intervention. In this study, the amount of level change was the difference in the level of infectious disease incidence before and after the intervention; the amount of slope change was the difference in the slope of the incidence of infectious diseases before and after the intervention and portrayed the change in the trends of infectious disease incidence before and after the intervention.


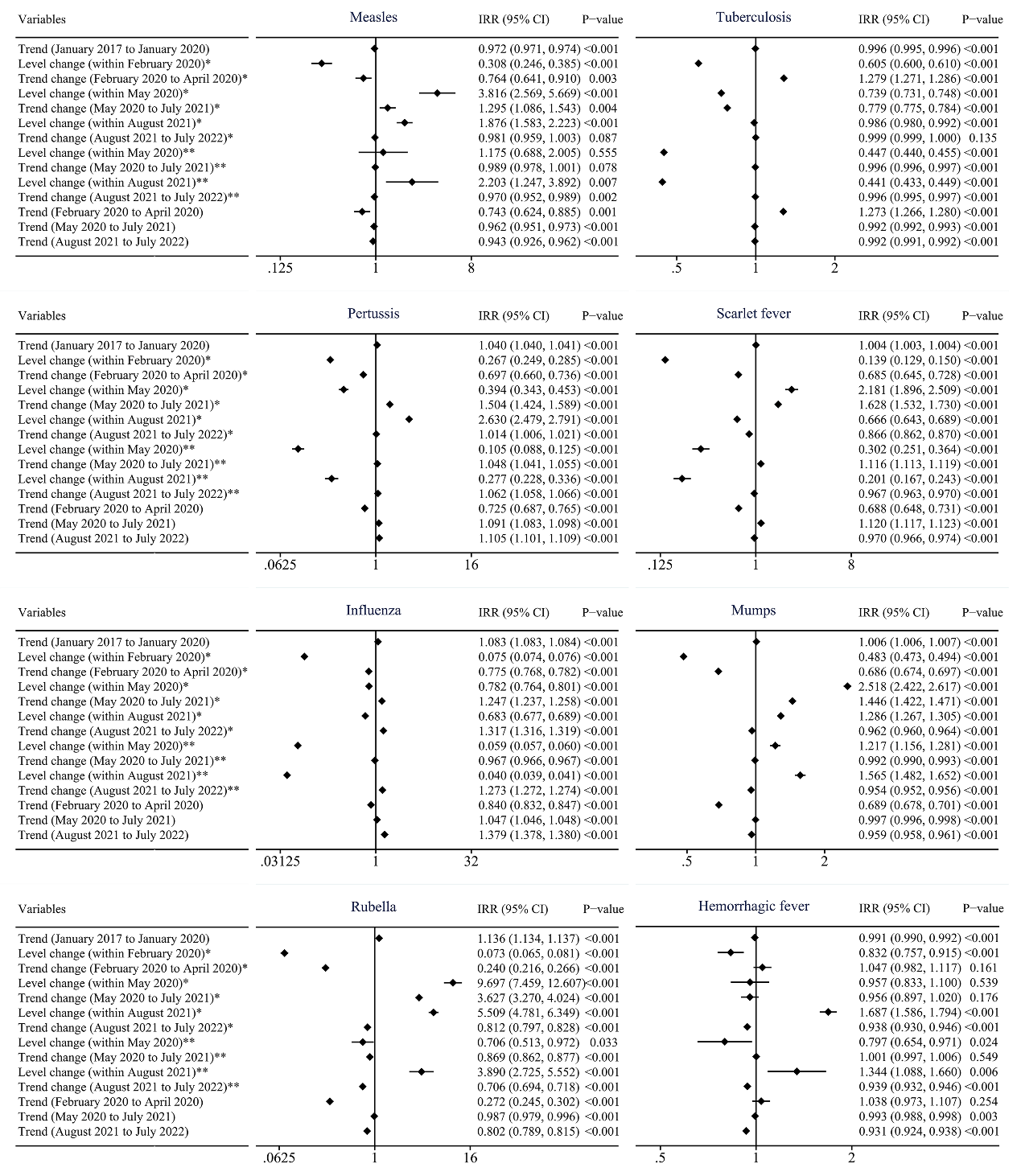


**Fig S1.** Immediate and long-term impact of “First-level-response”, “Normalized-control” and “Dynamic-zero-COVID” on respiratory-transmitted and vector-borne diseases. IRR: incidence rate ratio; CI: confidence interval. * indicates using the last intervention period as a reference; ** indicates using the no-COVID-19 period as a reference.


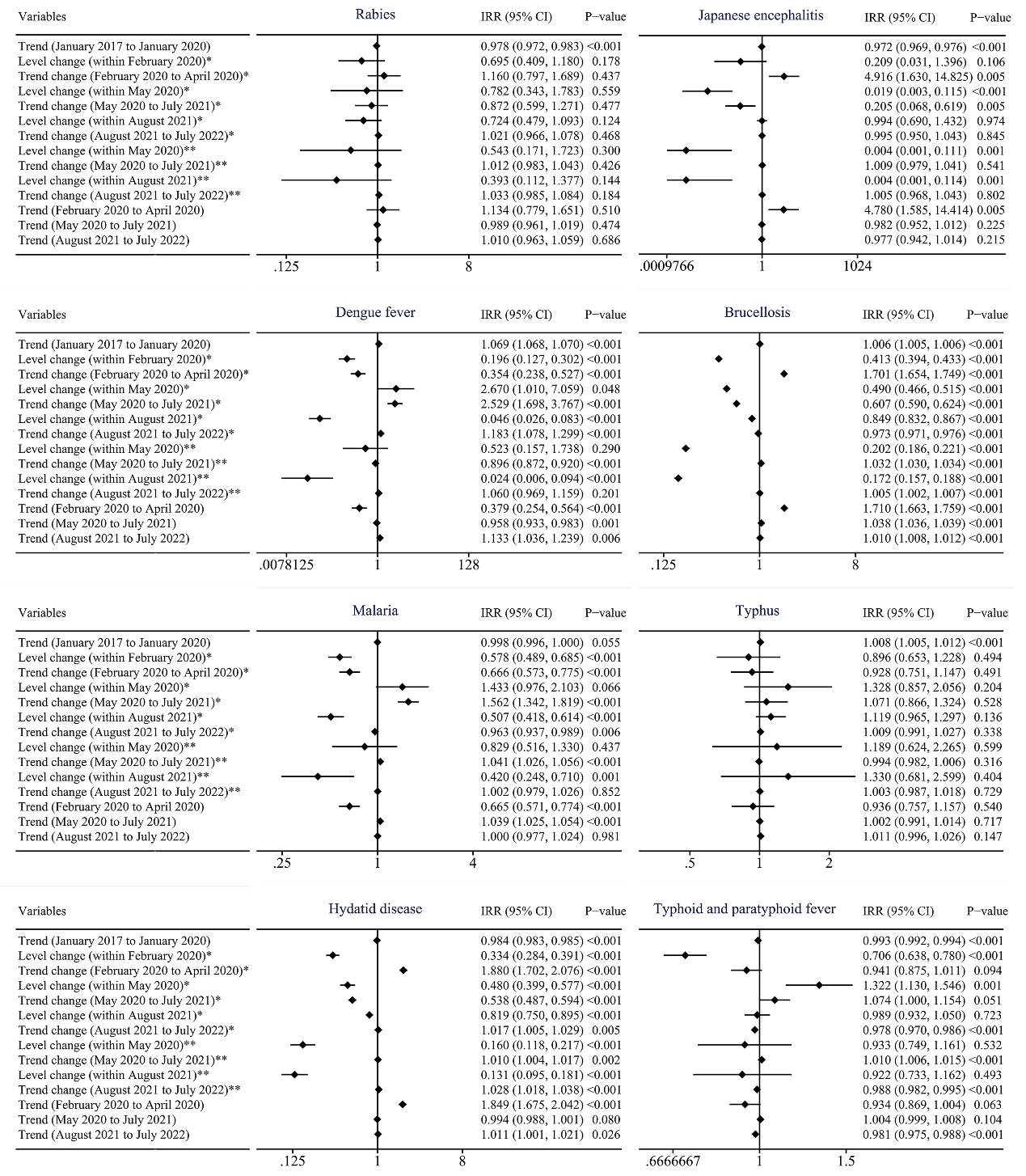


**Fig S2.** Immediate and long-term impact of “First-level-response”, “Normalized-control” and “Dynamic-zero-COVID” on vector-borne and gastrointestinal diseases. IRR: incidence rate ratio; CI: confidence interval. * indicates using the last intervention period as a reference; ** indicates using the no-COVID-19 period as a reference.


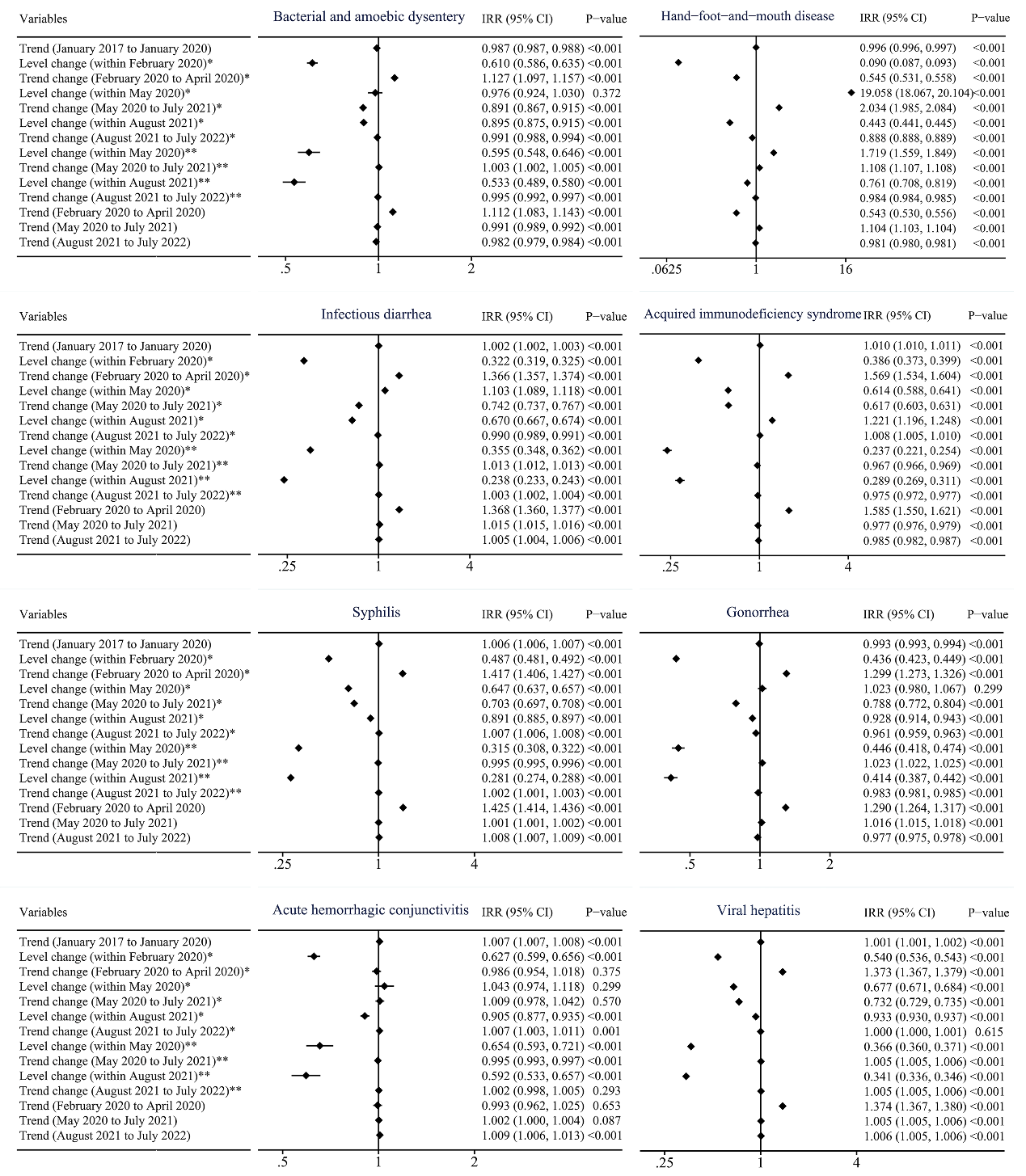
**Fig S3.** Immediate and long-term impact of “First-level-response”, “Normalized-control” and “Dynamic-zero-COVID” on other infectious diseases. IRR: incidence rate ratio; CI: confidence interval. * indicates using the last intervention period as a reference; ** indicates using the no-COVID-19 period as a reference.

**Table S1.** Characteristics of First-level-response, Normalized-control and in Dynamic-COVID-zero China from 2020 to 2022.

| **Strategies** | **First-level-response** | **Normalized-control** | **Dynamic-COVID-zero** |
| --- | --- | --- | --- |
| **Periods** | January 2020 to April 2020 | May 2020 to July 2021 | August 2021 to November 2022 |
| **General requirements** | Control the COVID-19 epidemic and extinguish the COVID-19 outbreaks everywhere | To strictly prevent the import of COVID-19 cases from abroad and control the COVID-19 outbreaks within 2 to 3 incubation periods | To balance the prevention and control of COVID-19 with socioeconomic stability and control the COVID-19 outbreak within 1 incubation period |
| **Stringency** | To implement the most stringent and numerous NPIs and comprehensively restrict population mobility and social distance | To implement the low-strict NPIs, people's lives are gradually returning to normal | The implementation of NPIs is more rapid and precise, which aimed to decrease the number of populations and geographic regions affected by NPIs |
| **NPIs** | National lockdown, individual movement restrictions, public transportation restrictions, border restrictions, travel restrictions, closure of educational institutions, mass gathering cancellation, isolation of cases and close contacts, public education, etc. | Widespread use of trip codes, health codes, and nucleic acid test reports, restrictions on the movement of people across regions, implementation of strict isolation measures (14 days of intensive isolation and 7 days of home isolation for close contacts and entrants, 7 days of intensive isolation for close contacts of close contacts), lockdown of schools, restrictions on mass gatherings, increased availability of PPE, environmental disinfection, lockdown of large areas in the cities with COVID-19 outbreaks, etc. | Continued use of trip codes, health codes, and nucleic acid testing, increasing availability of PPE, delineation of COVID-19 risk areas, mass nucleic acid testing, implementation of low-strict isolation measures (7 days of intensive isolation and 3 days of home isolation for entrants and close contacts and 7 days of home isolation for close contacts of close contacts), temporary lockdown of specific residential buildings or communities in areas with COVID-19 outbreaks, etc. |
| **Number of COVID-19 cases** | 82, 549 | 10, 131 | 229, 693 |

NPIs, non-pharmacological interventions; PPE, personal protective equipment.

**Table S2.** Immediate and long-term impact of “First-level-response”, “Normalized-control” and “Dynamic-zero-COVID” on measles, tuberculosis, pertussis, and scarlet fever in China, January 2015- July 2022.

| **Variable** | **Measles** | | **Tuberculosis** | | **Pertussis** | | **Scarlet fever** | |
| --- | --- | --- | --- | --- | --- | --- | --- | --- |
|  | **IRR (95% CI)** | ***P*-value** | **IRR (95% CI)** | ***P*-value** | **IRR (95% CI)** | ***P*-value** | **IRR (95% CI)** | ***P*-value** |
| **Taking the last intervention period as a reference, changes in level and trend during each period** | | | | | | | | |
| Trend before the first intervention | 0.940 (0.940 to 0.941) | <0.001 | 0.998 (0.997 to 0.998) | <0.001 | 1.038 (1.038 to 1.039) | <0.001 | 1.005 (1.005 to 1.005) | <0.001 |
| Level change with the first intervention | 0.515 (0.413 to 0.643) | <0.001 | 0.580 (0.576 to 0.585) | <0.001 | 0.276 (0.258 to 0.295) | <0.001 | 0.134 (0.124 to 0.145) | <0.001 |
| Trend change with the first intervention | 0.742 (0.622 to 0.884) | 0.001 | 1.278 (1.271 to 1.285) | <0.001 | 0.706 (0.669 to 0.746) | <0.001 | 0.690 (0.649 to 0.733) | <0.001 |
| Level change with the second intervention | 5.418 (3.647 to 8.047) | <0.001 | 0.749 (0.740 to 0.758) | <0.001 | 0.381 (0.332 to 0.438) | <0.001 | 2.232 (1.940 to 2.567) | <0.001 |
| Trend change with the second intervention | 1.369 (1.148 to 1.633) | <0.001 | 0.778 (0.774 to 0.782) | <0.001 | 1.488 (1.408 to 1.571) | <0.001 | 1.613 (1.518 to 1.714) | <0.001 |
| Level change with the third intervention | 3.326 (2.819 to 3.924) | <0.001 | 0.990 (0.984 to 0.995) | 0.001 | 2.605 (2.456 to 2.763) | <0.001 | 0.701 (0.678 to 0.725) | <0.001 |
| Trend change with the third intervention | 0.919 (0.900 to 0.940) | <0.001 | 1.000 (0.999 to 1.000) | 0.438 | 1.014 (1.007 to 1.022) | <0.001 | 0.864 (0.859 to 0.868) | <0.001 |
| **Taking the pre-COVID-19 period (from** **January 2015 to January 2020) as a reference, changes in level and trend during each period** | | | | | | | | |
| Level change with the second intervention | 2.791 (1.634 to 4.767) | <0.001 | 0.435 (0.427 to 0.442) | <0.001 | 0.105 (0.088 to 0.126) | <0.001 | 0.299 (0.249 to 0.360) | <0.001 |
| Trend change with the second intervention | 1.015 (1.004 to 1.027) | 0.007 | 0.994 (0.994 to 0.995) | <0.001 | 1.051 (1.044 to 1.058) | <0.001 | 1.112 (1.109 to 1.115) | <0.001 |
| Level change with the third intervention | 9.284 (5.267 to 16.392) | <0.001 | 0.430 (0.422 to 0.438) | <0.001 | 0.274 (0.226 to 0.333) | <0.001 | 0.210 (0.174 to 0.253) | <0.001 |
| Trend change with the third intervention | 0.933 (0.916 to 0.951) | <0.001 | 0.994 (0.993 to 0.994) | <0.001 | 1.066 (1.062 to 1.070) | <0.001 | 0.961 (0.957 to 0.964) | <0.001 |
| **Estimated trend during each period** | | | | | | | | |
| Trend after the first intervention | 0.697 (0.585 to 0.831) | <0.001 | 1.274 (1.267 to 1.281) | <0.001 | 0.733 (0.695 to 0.774) | <0.001 | 0.693 (0.652 to 0.736) | <0.001 |
| Trend after the second intervention | 0.955 (0.944 to 0.965) | <0.001 | 0.992 (0.991 to 0.992) | <0.001 | 1.091 (1.084 to 1.098) | <0.001 | 1.118 (1.115 to 1.121) | <0.001 |
| Trend after the third intervention | 0.878 (0.861 to 0.894) | <0.001 | 0.991 (0.991 to 0.992) | <0.001 | 1.106 (1.102 to 1.110) | <0.001 | 0.965 (0.962 to 0.969) | <0.001 |

IRR: incidence rate ratio; CI: confidence interval.

**Table S3.** Immediate and long-term impact of “First-level-response”, “Normalized-control” and “Dynamic-zero-COVID” on influenza, mumps, rubella, and hemorrhagic fever in China, January 2015- July 2022.

| **Variable** | **Influenza** | | **Mumps** | | **Rubella** | | **Hemorrhagic fever** | |
| --- | --- | --- | --- | --- | --- | --- | --- | --- |
|  | **IRR (95% CI)** | ***P*-value** | **IRR (95% CI)** | ***P*-value** | **IRR (95% CI)** | ***P*-value** | **IRR (95% CI)** | ***P*-value** |
| **Taking the last intervention period as a reference, changes in level and trend during each period** | | | | | | | | |
| Trend before the first intervention | 1.071 (1.071 to 1.071) | <0.001 | 1.011 (1.010 to 1.011) | <0.001 | 1.040 (1.039 to 1.040) | <0.001 | 1.000 (0.999 to 1.000) | 0.267 |
| Level change with the first intervention | 0.081 (0.081 to 0.082) | <0.001 | 0.437 (0.427 to 0.446) | <0.001 | 0.175 (0.157 to 0.195) | <0.001 | 0.655 (0.597 to 0.718) | <0.001 |
| Trend change with the first intervention | 0.768 (0.762 to 0.775) | <0.001 | 0.699 (0.687 to 0.710) | <0.001 | 0.313 (0.283 to 0.347) | <0.001 | 1.059 (0.993 to 1.130) | 0.081 |
| Level change with the second intervention | 0.846 (0.826 to 0.866) | <0.001 | 2.448 (2.355 to 2.545) | <0.001 | 6.906 (5.322 to 8.963) | <0.001 | 1.041 (0.907 to 1.196) | 0.565 |
| Trend change with the second intervention | 1.273 (1.262 to 1.284) | <0.001 | 1.412 (1.388 to 1.436) | <0.001 | 3.036 (2.740 to 3.365) | <0.001 | 0.935 (0.877 to 0.998) | 0.043 |
| Level change with the third intervention | 0.676 (0.670 to 0.682) | <0.001 | 1.307 (1.287 to 1.326) | <0.001 | 3.859 (3.343 to 4.454) | <0.001 | 1.780 (1.695 to 1.911) | <0.001 |
| Trend change with the third intervention | 1.320 (1.318 to 1.321) | <0.001 | 0.961 (0.959 to 0.963) | <0.001 | 0.846 (0.830 to 0.863) | <0.001 | 0.933 (0.925 to 0.941) | <0.001 |
| **Taking the pre-COVID-19 period (from January 2015 to January 2020) as a reference, changes in level and trend during each period** | | | | | | | | |
| Level change with the second intervention | 0.069 (0.067 to 0.071) | <0.001 | 1.070 (1.016 to 1.126) | 0.010 | 1.209 (0.881 to 1.660) | 0.241 | 0.682 (0.560 to 0.830) | <0.001 |
| Trend change with the second intervention | 0.978 (0.977 to 0.979) | <0.001 | 0.986 (0.985 to 0.987) | <0.001 | 0.951 (0.942 to 0.959) | <0.001 | 0.991 (0.986 to 0.995) | <0.001 |
| Level change with the third intervention | 0.047 (0.045 to 0.048) | <0.001 | 1.398 (1.324 to 1.475) | <0.001 | 4.664 (3.272 to 6.650) | <0.001 | 1.227 (0.994 to 1.514) | 0.057 |
| Trend change with the third intervention | 1.290 (1.290 to 1.291) | <0.001 | 0.948 (0.946 to 0.949) | <0.001 | 0.805 (0.791 to 0.818) | <0.001 | 0.925 (0.918 to 0.931) | <0.001 |
| **Estimated trend during each period** | | | | | | | | |
| Trend after the first intervention | 0.823 (0.816 to 0.830) | <0.001 | 0.706 (0.694 to 0.718) | <0.001 | 0.325 (0.294 to 0.361) | <0.001 | 1.059 (0.993 to 1.129) | 0.082 |
| Trend after the second intervention | 1.047 (1.046 to 1.048) | <0.001 | 0.997 (0.996 to 0.998) | <0.001 | 0.988 (0.979 to 0.997) | 0.010 | 0.990 (0.986 to 0.995) | <0.001 |
| Trend after the third intervention | 1.382 (1.381 to 1.382) | <0.001 | 0.958 (0.956 to 0.960) | <0.001 | 0.836 (0.822 to 0.851) | <0.001 | 0.924 (0.918 to 0.931) | <0.001 |

IRR: incidence rate ratio; CI: confidence interval.

**Table S4.** Immediate and long-term impact of “First-level-response”, “Normalized-control” and “Dynamic-zero-COVID” on rabies, Japanese encephalitis, dengue fever, and brucellosis in China, January 2015- July 2022.

| **Variable** | **Rabies** | | **Japanese encephalitis** | | **Dengue fever** | | **Brucellosis** | |
| --- | --- | --- | --- | --- | --- | --- | --- | --- |
|  | **IRR (95% CI)** | ***P*-value** | **IRR (95% CI)** | ***P*-value** | **IRR (95% CI)** | ***P*-value** | **IRR (95% CI)** | ***P*-value** |
| **Taking the last intervention period as a reference, changes in level and trend during each period** | | | | | | | | |
| Trend before the first intervention | 0.980 (0.978 to 0.982) | <0.001 | 0.998 (0.997 to 0.999) | 0.037 | 1.049 (1.048 to 1.049) | <0.001 | 0.993 (0.993 to 0.994) | <0.001 |
| Level change with the first intervention | 0.693 (0.415 to 1.156) | 0.160 | 0.129 (0.020 to 0.855) | 0.034 | 0.208 (0.135 to 0.320) | <0.001 | 0.529 (0.506 to 0.554) | <0.001 |
| Trend change with the first intervention | 1.177 (0.812 to 1.705) | 0.390 | 3.969 (1.323 to 11.913) | 0.014 | 0.393 (0.264 to 0.585) | <0.001 | 1.684 (1.638 to 1.732) | <0.001 |
| Level change with the second intervention | 0.703 (0.312 to 1.585) | 0.396 | 0.036 (0.006 to 0.210) | <0.001 | 2.429 (0.916 to 6.438) | 0.074 | 0.496 (0.472 to 0.521) | <0.001 |
| Trend change with the second intervention | 0.859 (0.592 to 1.245) | 0.422 | 0.246 (0.082 to 0.737) | 0.012 | 2.326 (1.560 to 3.469) | <0.001 | 0.620 (0.603 to 0.638) | <0.001 |
| Level change with the third intervention | 0.714 (0.475 to 1.073) | 0.105 | 1.133 (0.787 to 1.630) | 0.503 | 0.044 (0.025 to 0.081) | <0.001 | 0.866 (0.849 to 0.884) | <0.001 |
| Trend change with the third intervention | 1.021 (0.967 to 1.078) | 0.455 | 0.990 (0.944 to 1.038) | 0.682 | 1.197 (1.090 to 1.316) | <0.001 | 0.971 (0.968 to 0.973) | <0.001 |
| **Taking the pre-COVID-19 period (from January 2015 to January 2020) as a reference, changes in level and trend during each period** | | | | | | | | |
| Level change with the second intervention | 0.487 (0.156 to 1.519) | 0.215 | 0.005 (0.000 to 0.126) | 0.001 | 0.504 (0.151 to 1.684) | 0.266 | 0.263 (0.241 to 0.286) | <0.001 |
| Trend change with the second intervention | 1.010 (0.981 to 1.040) | 0.493 | 0.975 (0.946 to 1.005) | 0.097 | 0.913 (0.888 to 0.939) | <0.001 | 1.045 (1.043 to 1.046) | <0.001 |
| Level change with the third intervention | 0.348 (0.101 to 1.197) | 0.094 | 0.005 (0.000 to 0.148) | 0.002 | 0.022 (0.006 to 0.088) | <0.001 | 0.227 (0.208 to 0.248) | <0.001 |
| Trend change with the third intervention | 1.031 (0.984 to 1.081) | 0.194 | 0.965 (0.930 to 1.002) | 0.064 | 1.094 (0.999 to 1.197) | 0.052 | 1.014 (1.012 to 1.017) | <0.001 |
| **Estimated trend during each period** | | | | | | | | |
| Trend after the first intervention | 1.153 (0.796 to 1.671) | 0.451 | 3.963 (1.320 to 11.893) | 0.014 | 0.412 (0.276 to 0.613) | <0.001 | 1.673 (1.627 to 1.720) | <0.001 |
| Trend after the second intervention | 0.990 (0.962 to 1.019) | 0.511 | 0.973 (0.944 to 1.003) | 0.077 | 0.958 (0.932 to 0.984) | 0.002 | 1.038 (1.036 to 1.039) | <0.001 |
| Trend after the third intervention | 1.011 (0.965 to 1.059) | 0.642 | 0.964 (0.928 to 1.000) | 0.052 | 1.147 (1.048 to 1.255) | 0.003 | 1.008 (1.006 to 1.010) | <0.001 |

IRR: incidence rate ratio; CI: confidence interval.

**Table S5.** Immediate and long-term impact of “First-level-response”, “Normalized-control” and “Dynamic-zero-COVID” on malaria, typhus, hydatid disease, and typhoid and paratyphoid fever in China, January 2015- July 2022.

| **Variable** | **Malaria** | | **Typhus** | | **Hydatid disease** | | **Typhoid and paratyphoid fever** | |
| --- | --- | --- | --- | --- | --- | --- | --- | --- |
|  | **IRR (95% CI)** | ***P*-value** | **IRR (95% CI)** | ***P*-value** | **IRR (95% CI)** | ***P*-value** | **IRR (95% CI)** | ***P*-value** |
| **Taking the last intervention period as a reference, changes in level and trend during each period** | | | | | | | | |
| Trend before the first intervention | 0.995 (0.994 to 0.996) | <0.001 | 0.994 (0.992 to 0.995) | <0.001 | 1.001 (1.001 to 1.002) | <0.001 | 0.996 (0.995 to 0.996) | <0.001 |
| Level change with the first intervention | 0.640 (0.541 to 0.759) | <0.001 | 1.002 (0.736 to 1.364) | 0.992 | 0.223 (0.190 to 0.261) | <0.001 | 0.657 (0.596 to 0.725) | <0.001 |
| Trend change with the first intervention | 0.679 (0.584 to 0.791) | <0.001 | 0.970 (0.785 to 1.198) | 0.776 | 1.976 (1.788 to 2.185) | <0.001 | 0.936 (0.871 to 1.005) | 0.069 |
| Level change with the second intervention | 1.293 (0.881 to 1.897) | 0.189 | 1.404 (0.909 to 2.167) | 0.126 | 0.455 (0.377 to 0.549) | <0.001 | 1.385 (1.185 to 1.619) | <0.001 |
| Trend change with the second intervention | 1.532 (1.315 to 1.784) | <0.001 | 1.037 (0.839 to 1.281) | 0.739 | 0.501 (0.453 to 0.554) | <0.001 | 1.076 (1.001 to 1.156) | 0.046 |
| Level change with the third intervention | 0.607 (0.502 to 0.735) | <0.001 | 1.170 (1.013 to 1.353) | 0.033 | 0.791 (0.720 to 0.869) | <0.001 | 1.011 (0.954 to 1.073) | 0.706 |
| Trend change with the third intervention | 0.946 (0.921 to 0.972) | <0.001 | 1.008 (0.990 to 1.026) | 0.403 | 1.029 (1.016 to 1.042) | <0.001 | 0.977 (0.969 to 0.984) | <0.001 |
| **Taking the pre-COVID-19 period (from January 2015 to January 2020) as a reference, changes in level and trend during each period** | | | | | | | | |
| Level change with the second intervention | 0.828 (0.517 to 1.327) | 0.433 | 1.406 (0.741 to 2.669) | 0.297 | 0.101 (0.074 to 0.137) | <0.001 | 0.910 (0.732 to 1.132) | 0.399 |
| Trend change with the second intervention | 1.040 (1.027 to 1.055) | <0.001 | 1.005 (0.994 to 1.017) | 0.364 | 0.989 (0.983 to 0.996) | 0.002 | 1.007 (1.002 to 1.011) | 0.003 |
| Level change with the third intervention | 0.503 (0.298 to 0.848) | 0.010 | 1.646 (0.846 to 3.200) | 0.142 | 0.080 (0.058 to 0.111) | <0.001 | 0.921 (0.732 to 1.158) | 0.480 |
| Trend change with the third intervention | 0.985 (0.962 to 1.008) | 0.191 | 1.013 (0.998 to 1.028) | 0.081 | 1.018 (1.007 to 1.029) | 0.001 | 0.983 (0.977 to 0.990) | <0.001 |
| **Estimated trend during each period** | | | | | | | | |
| Trend after the first intervention | 0.676 (0.581 to 0.786) | <0.001 | 0.964 (0.780 to 1.190) | 0.732 | 1.979 (1.790 to 2.188) | <0.001 | 0.932 (0.868 to 1.001) | 0.053 |
| Trend after the second intervention | 1.035 (1.021 to 1.049) | <0.001 | 0.999 (0.988 to 1.010) | 0.864 | 0.991 (0.984 to 0.997) | 0.007 | 1.002 (0.998 to 1.007) | 0.263 |
| Trend after the third intervention | 0.979 (0.957 to 1.002) | 0.079 | 1.007 (0.992 to 1.021) | 0.364 | 1.019 (1.009 to 1.030) | <0.001 | 0.979 (0.973 to 0.985) | <0.001 |

IRR: incidence rate ratio; CI: confidence interval.

**Table S6.** Immediate and long-term impact of “First-level-response”, “Normalized-control” and “Dynamic-zero-COVID” on bacterial and amoebic dysentery, hand-foot-and-mouth disease, infectious diarrhea, and acquired immunodeficiency syndrome in China, January 2015- July 2022.

| **Variable** | **Bacterial and amoebic dysentery** | | **Hand-foot-and-mouth disease** | | **Infectious diarrhea** | | **Acquired immunodeficiency syndrome** | |
| --- | --- | --- | --- | --- | --- | --- | --- | --- |
|  | **IRR (95% CI)** | ***P*-value** | **IRR (95% CI)** | ***P*-value** | **IRR (95% CI)** | ***P*-value** | **IRR (95% CI)** | ***P*-value** |
| **Taking the last intervention period as a reference, changes in level and trend during each period** | | | | | | | | |
| Trend before the first intervention | 0.988 (0.988 to 0.989) | <0.001 | 0.998 (0.998 to 0.998) | <0.001 | 1.007 (1.007 to 1.007) | <0.001 | 1.007 (1.007 to 1.007) | <0.001 |
| Level change with the first intervention | 0.618 (0.594 to 0.643) | <0.001 | 0.084 (0.081 to 0.086) | <0.001 | 0.304 (0.302 to 0.307) | <0.001 | 0.568 (0.549 to 0.588) | <0.001 |
| Trend change with the first intervention | 1.115 (1.085 to 1.145) | <0.001 | 0.525 (0.513 to 0.538) | <0.001 | 1.366 (1.358 to 1.375) | <0.001 | 1.343 (1.313 to 1.373) | <0.001 |
| Level change with the second intervention | 0.964 (0.913 to 1.017) | 0.182 | 22.421 (21.255 to 23.651) | <0.001 | 1.053 (1.039 to 1.067) | <0.001 | 0.659 (0.630 to 0.688) | <0.001 |
| Trend change with the second intervention | 0.900 (0.876 to 0.925) | <0.001 | 2.093 (2.043 to 2.145) | <0.001 | 0.739 (0.734 to 0.743) | <0.001 | 0.729 (0.713 to 0.745) | <0.001 |
| Level change with the third intervention | 0.868 (0.849 to 0.887) | <0.001 | 0.530 (0.528 to 0.532) | <0.001 | 0.654 (0.651 to 0.657) | <0.001 | 1.159 (1.134 to 1.185) | <0.001 |
| Trend change with the third intervention | 0.993 (0.990 to 0.996) | <0.001 | 0.877 (0.876 to 0.877) | <0.001 | 0.991 (0.991 to 0.992) | <0.001 | 1.001 (0.998 to 1.004) | 0.365 |
| **Taking the pre-COVID-19 period (from January 2015 to January 2020) as a reference, changes in level and trend during each period** | | | | | | | | |
| Level change with the second intervention | 0.596 (0.549 to 0.646) | <0.001 | 1.877 (1.745 to 2.019) | <0.001 | 0.320 (0.314 to 0.326) | <0.001 | 0.374 (0.349 to 0.401) | <0.001 |
| Trend change with the second intervention | 1.004 (1.002 to 1.005) | <0.001 | 1.100 (1.099 to 1.100) | <0.001 | 1.009 (1.009 to 1.010) | <0.001 | 0.978 (0.977 to 0.980) | <0.001 |
| Level change with the third intervention | 0.517 (0.475 to 0.563) | <0.001 | 0.995 (0.925 to 1.070) | 0.893 | 0.210 (0.205 to 0.214) | <0.001 | 0.434 (0.403 to 0.467) | <0.001 |
| Trend change with the third intervention | 0.997 (0.994 to 0.999) | 0.009 | 0.964 (0.964 to 0.965) | <0.001 | 1.001 (1.000 to 1.001) | 0.079 | 0.980 (0.977 to 0.982) | <0.001 |
| **Estimated trend during each period** | | | | | | | | |
| Trend after the first intervention | 1.102 (1.073 to 1.132) | <0.001 | 0.524 (0.512 to 0.537) | <0.001 | 1.376 (1.367 to 1.384) | <0.001 | 1.352 (1.322 to 1.382) | <0.001 |
| Trend after the second intervention | 0.992 (0.990 to 0.993) | <0.001 | 1.097 (1.097 to 1.098) | <0.001 | 1.016 (1.016 to 1.017) | <0.001 | 0.985 (0.983 to 0.987) | <0.001 |
| Trend after the third intervention | 0.985 (0.983 to 0.988) | <0.001 | 0.962 (0.962 to 0.963) | <0.001 | 1.008 (1.007 to 1.008) | <0.001 | 0.986 (0.984 to 0.989) | <0.001 |

IRR: incidence rate ratio; CI: confidence interval.

**Table S7.** Immediate and long-term impact of “First-level-response”, “Normalized-control” and “Dynamic-zero-COVID” on syphilis, gonorrhea, acute hemorrhagic conjunctivitis, and viral hepatitis in China, January 2015- July 2022.

| **Variable** | **Syphilis** | | **Gonorrhea** | | **Acute hemorrhagic conjunctivitis** | | **Viral hepatitis** | |
| --- | --- | --- | --- | --- | --- | --- | --- | --- |
|  | **IRR (95% CI)** | ***P*-value** | **IRR (95% CI)** | ***P*-value** | **IRR (95% CI)** | ***P*-value** | **IRR (95% CI)** | ***P*-value** |
| **Taking the last intervention period as a reference, changes in level and trend during each period** | | | | | | | | |
| Trend before the first intervention | 1.005 (1.005 to 1.005) | <0.001 | 1.003 (1.003 to 1.003) | <0.001 | 1.004 (1.003 to 1.004) | <0.001 | 1.002 (1.002 to 1.002) | <0.001 |
| Level change with the first intervention | 0.551 (0.545 to 0.557) | <0.001 | 0.367 (0.356 to 0.378) | <0.001 | 0.663 (0.634 to 0.693) | <0.001 | 0.521 (0.518 to 0.525) | <0.001 |
| Trend change with the first intervention | 1.327 (1.317 to 1.337) | <0.001 | 1.288 (1.262 to 1.315) | <0.001 | 0.995 (0.964 to 1.027) | 0.777 | 1.344 (1.338 to 1.351) | <0.001 |
| Level change with the second intervention | 0.681 (0.671 to 0.692) | <0.001 | 1.038 (0.995 to 1.082) | 0.085 | 1.017 (0.950 to 1.089) | 0.630 | 0.727 (0.720 to 0.734) | <0.001 |
| Trend change with the second intervention | 0.753 (0.747 to 0.758) | <0.001 | 0.786 (0.770 to 0.802) | <0.001 | 1.003 (0.972 to 1.036) | 0.840 | 0.745 (0.742 to 0.749) | <0.001 |
| Level change with the third intervention | 0.896 (0.889 to 0.902) | <0.001 | 0.943 (0.929 to 0.958) | <0.001 | 0.903 (0.875 to 0.932) | <0.001 | 0.979 (0.975 to 0.983) | <0.001 |
| Trend change with the third intervention | 1.001 (1.000 to 1.002) | 0.013 | 0.960 (0.958 to 0.962) | <0.001 | 1.007 (1.003 to 1.011) | 0.001 | 0.996 (0.995 to 0.996) | <0.001 |
| **Taking the no-COVID-19 period (from January 2015 to January 2020) as a reference, changes in level and trend during each period** | | | | | | | | |
| Level change with the second intervention | 0.375 (0.367 to 0.384) | <0.001 | 0.381 (0.358 to 0.405) | <0.001 | 0.674 (0.612 to 0.743) | <0.001 | 0.379 (0.374 to 0.385) | <0.001 |
| Trend change with the second intervention | 0.999 (0.999 to 1.000) | 0.001 | 1.013 (1.011 to 1.014) | <0.001 | 0.999 (0.996 to 1.001) | 0.257 | 1.002 (1.002 to 1.002) | <0.001 |
| Level change with the third intervention | 0.336 (0.328 to 0.345) | <0.001 | 0.359 (0.337 to 0.383) | <0.001 | 0.609 (0.549 to 0.675) | <0.001 | 0.371 (0.366 to 0.377) | <0.001 |
| Trend change with the third intervention | 1.001 (0.999 to 1.001) | 0.465 | 0.972 (0.970 to 0.973) | <0.001 | 1.006 (1.002 to 1.009) | 0.001 | 0.998 (0.997 to 0.998) | <0.001 |
| **Estimated trend during each period** | | | | | | | | |
| Trend after the first intervention | 1.334 (1.324 to 1.344) | <0.001 | 1.292 (1.266 to 1.319) | <0.001 | 0.999 (0.968 to 1.031) | 0.955 | 1.347 (1.341 to 1.353) | <0.001 |
| Trend after the second intervention | 1.004 (1.003 to 1.005) | <0.001 | 1.016 (1.015 to 1.017) | <0.001 | 1.002 (1.000 to 1.005) | 0.038 | 1.004 (1.004 to 1.004) | <0.001 |
| Trend after the third intervention | 1.005 (1.004 to 1.006) | <0.001 | 0.975 (0.973 to 0.976) | <0.001 | 1.009 (1.006 to 1.013) | <0.001 | 1.000 (0.999 to 1.000) | 0.332 |

IRR: incidence rate ratio; CI: confidence interval.
